# A rare case of pediatric cutaneous bullous mastocytosis

**DOI:** 10.1016/j.jdcr.2024.11.041

**Published:** 2024-12-25

**Authors:** Jennifer Roux, Christine T. Pham, Katerina Yale, Nathan W. Rojek, Kenneth Linden, Linda Doan

**Affiliations:** a University of California, Irvine School of Medicine, Irvine, California; b Department of Dermatology, University of California, Irvine, Irvine, California

**Keywords:** bullous mastocytosis, cutaneous mastocytosis, mastocytosis, pediatric, Steven Johnson Syndrome, toxic epidermal necrolysis

## Introduction

Mastocytosis comprises a group of disorders characterized by an accumulation of mast cells in various organs. It is divided into cutaneous mastocytosis (CM) and systemic mastocytosis (SM). CM is the predominant form observed in children and there are multiple variants.^1^ Here, we present a case that was initially thought to be Stevens-Johnson Syndrome (SJS)/Toxic epidermal necrolysis (TEN), and later confirmed to be bullous mastocytosis (BM), an uncommon presentation of CM.

## Case report

A previously healthy 2-year-old female presented as a transfer for suspected SJS/TEN following an upper respiratory illness. She was treated with acetaminophen at home and subsequently experienced a seizure, leading to admission at an outside hospital where she received levetiracetam, vancomycin, and acyclovir. A few hours later, she developed tense blisters that evolved into diffuse desquamating lesions on her chest and back.

**Fig 1:**
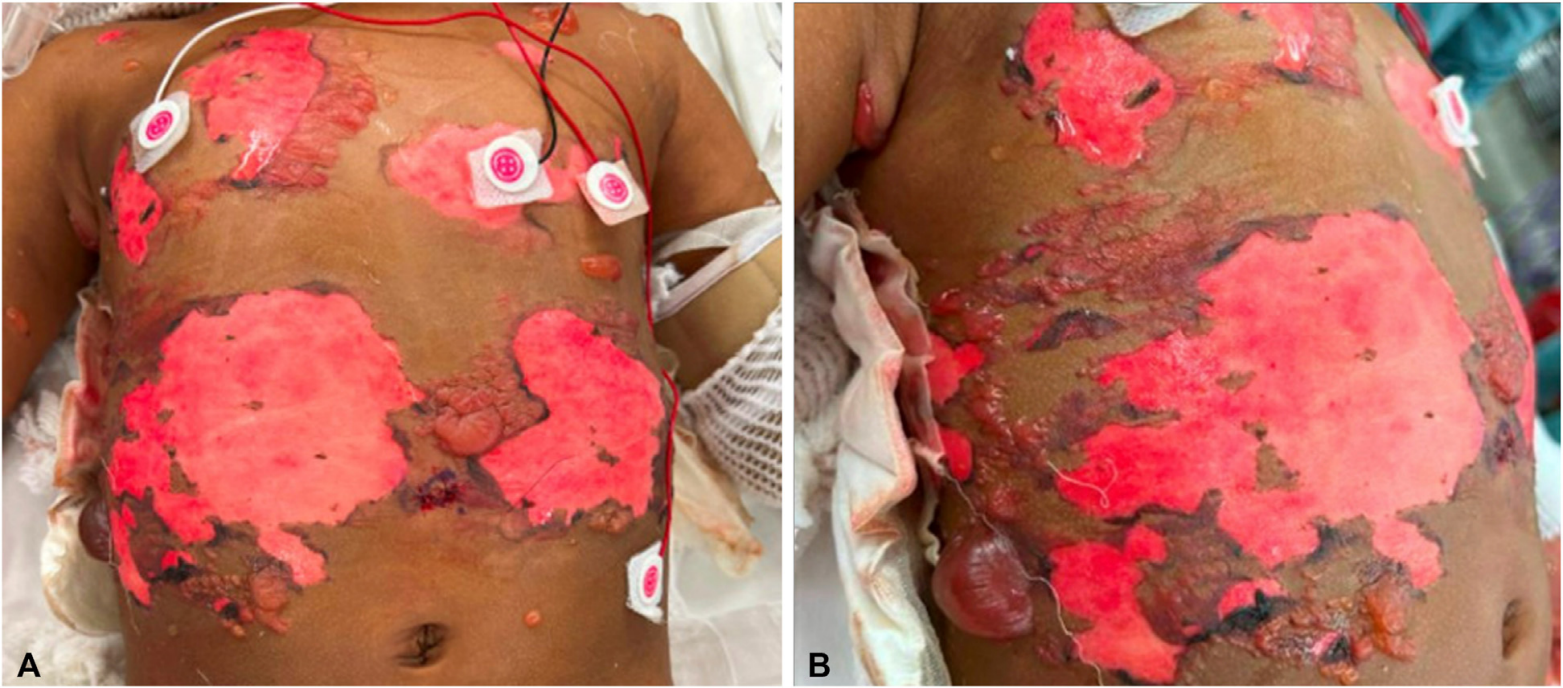
Two-year-old female on hospital day 3 with full-thickness desquamation and underlying erythema with surrounding tense bullae on the chest, back, and posterior neck, involving 20% body surface area (**A** and **B**).

Skin examination on hospital day 3 revealed full-thickness desquamation with underlying erythema and surrounding tense bullae on the chest, back, and neck, involving 20% of the patient’s body surface area (Fig 1). There was no mucosal or ocular involvement. An atypical presentation of SJS/TEN secondary to viral infection versus acetaminophen was favored, although these are uncommon triggers. Linear IgA bullous dermatosis, bullous drug eruption, and staphylococcal scalded skin syndrome were considered as well. Intravenous immunoglobulin infusions were initiated and punch biopsies for histologic and direct immunofluorescence studies were obtained.

**Fig 2:**
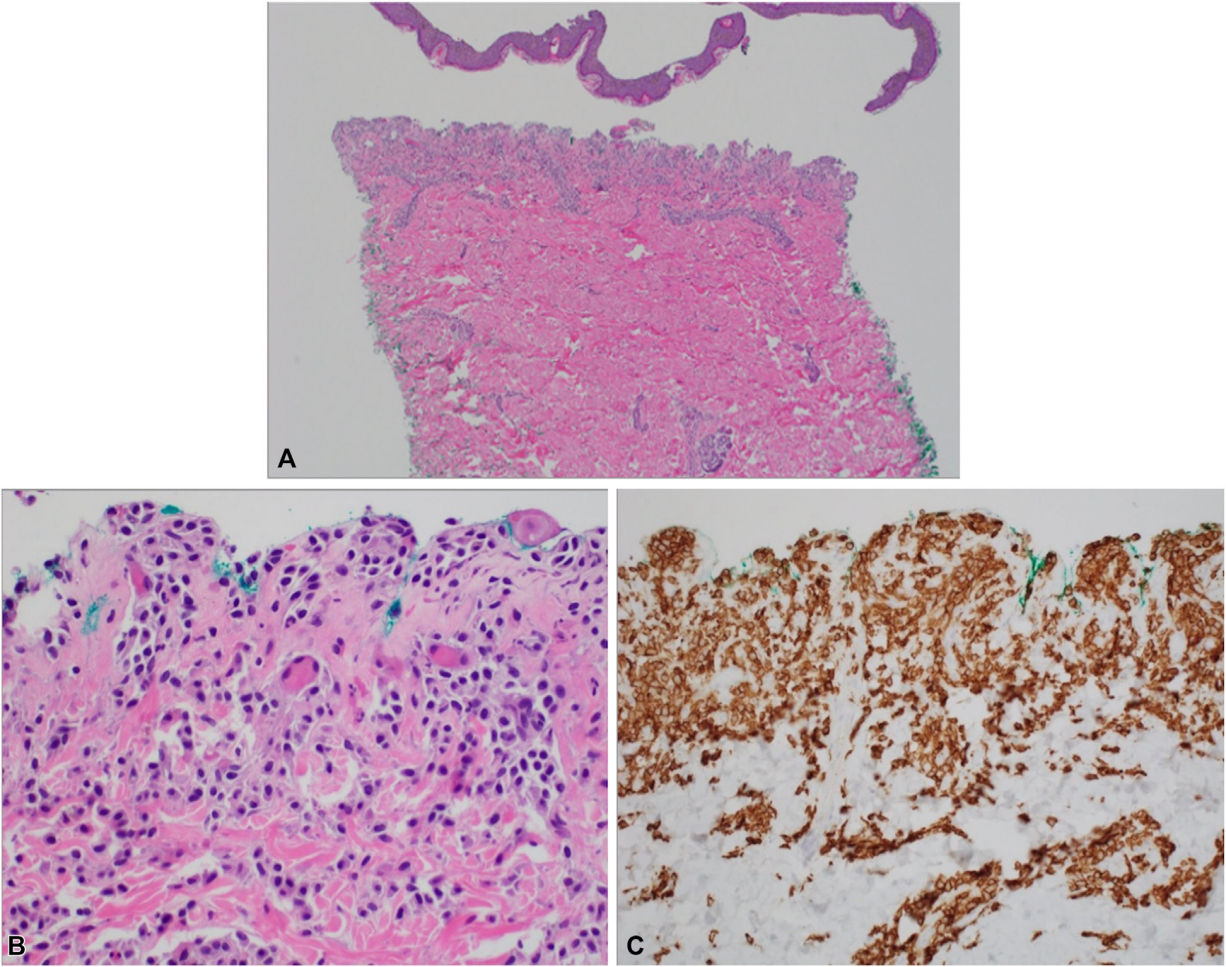
Hematoxylin and eosin, 4× (**A**) and 40× (**B**). Subepidermal split with numerous monomorphic cells in the upper dermis, resembling mast cells. CD117 stain, 40× (**C**) highlights a proliferation of mast cells in the upper dermis, confirming the diagnosis of mastocytosis.

Histology demonstrated a subepidermal blister with a dermal infiltrate of numerous round monomorphic cells (Fig 2). Immunohistochemical staining revealed expression of CD117 and tryptase (Fig 2), confirming mast cell lineage, and rendering the diagnosis of BM. Direct immunofluorescence studies were negative for an autoimmune blistering disorder. An elevated serum tryptase level of 29 ng/ml supported the diagnosis. Complete blood count with differential and a serum chemistry were unremarkable. Treatment began with 12 mg methylprednisolone IV daily for 1 week, along with cetirizine, famotidine, and topical clobetasol. The methylprednisolone was tapered to 10 mg for 4 days, then 5 mg for 4 days, and then it was discontinued.

The patient demonstrated dermatologic improvement just a few days after treatment was initiated, showing areas of re-epithelialization, reduced erythema, and no new bullae or ulcerations. By the 2-week mark, a skin examination revealed nearly complete re-epithelialization and resolution of bullae and ulcerations.

During the hospitalization, the patient had worsening anemia, prompting a consultation with hematology-oncology. However, due to her improving tryptase levels and benign abdominal ultrasound with no suggestion of organomegaly, further hematology-oncology workup was deferred to an outpatient basis. The patient ultimately did not require a bone marrow biopsy.

She was discharged on famotidine and cetirizine and her parents were advised on how to avoid triggers that could induce mast cell degranulation. The patient continued to show improvement and regression of skin lesions at follow-up visits.

## Discussion

The estimated prevalence of pediatric mastocytosis is 1-3 per 10,000, with the majority of cases being CM rather than SM.^2^ CM consist of 3 subtypes: maculopapular CM, cutaneous mastocytoma, and diffuse CM. BM is an exceptionally rare and severe presentation that can be associated with all subtypes of CM, often manifesting within 48 hours of birth, although cases have been reported in children up to 6 years old.^1^ BM has not been reported in adults.

The clinical presentation of BM can resemble that of SJS/TEN or other pediatric bullous conditions including staphylococcal scalded skin syndrome, linear IgA bullous dermatosis, epidermolysis bullosa, bullous impetigo, and bullous erythema multiforme.^2^ Physical examination may demonstrate skin thickening, blisters, erythema, and positive Darier’s sign.^1^ Systemic symptoms, such as pruritus, flushing, and gastrointestinal disturbances, may arise due to the release of histamine and other mast cell mediators into the bloodstream.^3^

Diagnosing mastocytosis requires a comprehensive evaluation and, for BM in particular, an experienced pathologist. The histologic findings can be misinterpreted as the mast cell infiltrate can be subtle and overlooked, and having a thorough histologic differential for a subepidermal blistering dermatitis is paramount. In this case, the histologic differential diagnosis included bullous pemphigoid and linear IgA. However, the negative direct immunofluorescence militated against an autoimmune blistering disorder, and the detection of mast cells via IHC confirmed the diagnosis of BM. A patient's history may uncover recent exposure to triggers of mast cell degranulation, including environmental factors or medications.^4^ Commonly reported medication culprits include antibiotics (fluoroquinolones, vancomycin, rifampin, amphotericin B), opioids, neuromuscular blocking agents, and acetylsalicylic acid.^5^ Vancomycin was the probable cause in our case, given its administration preceding the patient’s rash development and its known ability to induce histamine release from mast cells.^5^ Diagnostic assessments should also include a complete blood count with differential, serum chemistry, and serum tryptase.^1^

Serum tryptase levels exceeding 20 ng/mL, cytopenias, and organomegaly are concerning for SM.^3^ Our patient’s serum tryptase reached 29.5 ng/mL, and she experienced worsening anemia during admission, prompting evaluation for SM. Organomegaly was absent, and our patient’s serum tryptase levels normalized as lesions resolved. It has been noted that serum tryptase levels can be elevated in the presence of extensive skin lesions.^1^

In managing mastocytosis, the primary aim is to mitigate the release of mast cell mediators via avoidance of environmental and medication triggers.^6^ Symptomatic relief is an important therapeutic goal, often achievable with oral antihistamines and topical corticosteroids and emollients.^6^ Systemic corticosteroids can be considered for extensive lesions and recurrent blistering. For children with extensive skin involvement, a history of severe systemic symptoms or anaphylaxis, and markedly elevated serum tryptase levels, having auto injectable epinephrine is advisable.^6^

Although there is no curative treatment, most children with CM experience spontaneous resolution by adolescence.^6^ The prognosis for complete resolution in BM is less certain due to its rarity.^2^ In contrast to pediatric mastocytosis, adult-onset mastocytosis often involves multiorgan complications, carries a higher risk of anaphylaxis, and tends to have a persistent course.^7^

In summary, a thorough clinical evaluation, detailed history, and diagnostic tests are vital to differentiate BM from other blistering skin conditions, such as SJS/TEN, which can be life-threatening. Children diagnosed with CM typically have a favorable prognosis with rare progression to systemic involvement, especially with early diagnosis and proper management.

## Conflicts of interest

None disclosed.
